# Doing more with movement: constituting healthy publics in movement volunteering programmes

**DOI:** 10.1057/s41599-020-0473-9

**Published:** 2020-05-13

**Authors:** Emily Tupper, Sarah Atkinson, Tessa M. Pollard

**Affiliations:** 1 Department of Anthropology and The Institute for Medical Humanities, Durham University, Durham, UK; 2 Department of Geography and The Institute for Medical Humanities, Durham University, Durham, UK; 3 Department of Anthropology, Durham University, Durham, UK

## Abstract

The recent phenomenon of movement volunteering programmes is a form of ‘fitness philanthropy’ that combines exercise with volunteering in order for physical activity to generate a more widely shared set of benefits. These newest practices of fitness philanthropy radically rework both exercise and volunteering through the ways in which these come together and take place outdoors and in the everyday spaces of the street or community. The paper explores these new practices through the movement volunteering programme ‘GoodGym’, in relation to the concept of ‘healthy publics’. Fieldwork comprised ethnography, including participant observation, interviews, go-along interviews, conversations, photography and an end of fieldwork discussion workshop. We focus on the experiences of three different constituencies in GoodGym: the volunteers; the participants and passers-by; the space and atmosphere. The formation of these dynamic, multiple and shifting healthy publics emerge through the complex intersections of several processes. We draw particular attention to the centrality in the new fitness philanthropy practices of visibility and spectacle, sociality and merging mobilities in constituting healthy publics.

## Introduction

In this paper, we explore the simultaneous ways in which people become active while maintaining and transforming physical and relational aspects of the public through the recent phenomenon of movement volunteering programmes, the newest version of everyday ‘fitness philanthropy’ (Palmer and Dwyer, 2019). We propose that these new practices engage, participate with and influence public spaces and those in them, and, through their visibility and spectacle, sociality and a merging of embodied mobilities, bring into being improvised and everyday ‘healthy publics’ (Hinchliffe et al., 2018). The paper draws on research with a movement volunteering programme designed to bring people together and, at the same time, provide inclusive and collective experiences of movement and philanthropic activity. We argue that these programmes constitute particular opportunities for the formation of healthy publics through new kinds of public participation, engagement and social contract, in which health becomes a collective process and through which ‘moving together’ in everyday spaces is imagined and realised to mutual benefit.

The concept of ‘Healthy Publics’, the theme of this collection, is an offer to challenge how we think about publics and their position in our research and practice (Hinchliffe et al., 2018). This rich reimagining of practice includes, among other things, relocating our attention onto processes, relations, experience and experiments. Publics are ‘in-the-making’, not pre-given, and may be constituted as intentional collaborative spaces of discourse or as collectives of interests and affects coalescing around particular health concerns (Healey, 2019; Lünenborg, 2020; Mahoney and Stephansen, 2017; Peltola et al., 2018) and may include new connectivities from online and digitally networked publics (Lünenborg and Raetzsch, 2017; Stephansen, 2016). A healthy public is also likely to be multi-species in composition even if engagement and participation may take radically different modes (Rock, 2017). The framing offers a flexible conceptual space for thinking about broader relations of engagement and equitable collaborations. Nonetheless, examples in the literature tend to comprise self-defined constituencies, to focus on voice in terms of expertise and experience and to explore practices in relation to an intentional purpose. We take the concept in complementary directions onto healthy publics that are not of necessity self-defining and that have effects beyond the intended scope of an experimental intervention. We do this through an ethnographic case study of GoodGym, an organisation exemplifying the emergence of movement volunteering, a version of fitness philanthropy (Palmer, 2018) and which combines embodied performances of health through physical activity and the moral imaginings of public through voluntary action.

## Physical activity and the running boom

How, where and why people are physically active has changed greatly in the UK over the last 50 years. Work practices have become increasingly sedentary with the result that physical activity mostly occurs during our travel or our leisure time, and quite often takes place in public or ‘third’ spaces in which people engage and relate to one another (Amin, 2008; Latham and Layton, 2019; Thang, 2015). We have also witnessed a shift away from the hegemony of sport towards forms of physical activity that are personally adjustable in that ‘there are more activities to choose from and individual flexibility to determine the content of activities’ (Tainio, 2018, p. 3). Our physical activity has, thus, become understood as ‘exercise’ and with an objective of improvement or maintenance of physical fitness (Caspersen et al., 1985). These shifts accompany the considerable concern about low levels of physical activity in the population as a whole (Kohl et al., 2012; Pratt et al., 2019) and the public health campaigns that promote moderate to vigorous physical activity to enhance health. For example, the public health campaign *Active 10* encourages people to ‘speed up’ their walking and to ‘turn walking into exercise’ (https://www.nhs.uk/oneyou/active10/home).

One form of physical activity that has rapidly expanded within this context is running. Running takes exercise outdoors and onto the streets (Scheerder et al., 2015). In an outdoors physical activity, to be active is of necessity a form of participation, even if the exercise is undertaken alone, as it demands engagement with the embedding environment of public space and, as such, folds practice, movement and participation into one another (Cook et al., 2016; Hitchings and Latham, 2017). Running, then, constitutes both a responsible approach to maintaining health and fitness and a public practice. Running has also become an important tool through which non-state actors design and promote physical activity and movement both to specific groups and at population level. The exponential growth of parkrun (a free, weekly, timed 5 k event in local parks) has made running a mass-participation activity while *Achilles International* has engaged with running as a way,

‘to empower people with all types of disabilities to participate in mainstream running events in order to promote personal achievement’ https://www.achillesinternational.org/.

In a similar vein, *The Running Charity* uses running as a way to build resilience, confidence, and self-esteem for 16–25-year-olds who are homeless or at risk of homelessness https://www.therunningcharity.org/. The growth of these targeted activities reveals the potential of running as an embodied, emotional, and disciplinary practice for catalysing improvements in individual health and life circumstances.

The health potential of running is inseparable from the participation potential of different social groups and in different locations. While running is considered a ‘democratic’ activity due to its ease, simplicity, low cost (Latham and Hitchings, 2016, p. 506) and even its ‘humanness’ (Whelan, 2012), there are at least three aspects to running that may undermine this argument. A social practice is reliant on the groups of people it recruits and how this practice is carried culturally (Blue et al., 2014). This means that what is normal, acceptable, or safe within a group of people or within a given place is important for the survival and persistence of a practice. These implicit or explicit rules will affect the competencies, materials and meanings of a practice. In terms of competencies, while running may not seem to demand any particular skill, those taking up running must have at least some level of mobility and usually define a programme to build their confidence and strength as in the *‘Couch to 5 k’* movement (https://www.nhs.uk/live-well/exercise/get-running-with-couch-to-5k/). The materialities of equipment, such as shoes and clothes, and environments, such as weather, surfaces and security, for running are also potential barriers to participation (Gattrell, 2013; Schwanen et al., 2012). Although switching between indoor and outdoor running may moderate seasonal weather changes they are also different social practices, and valued in distinct ways by practitioners (Hitchings and Latham, 2016). Concerns for safety are more absolute as a barrier and likely to be strongly associated with socio-economic gradients. Running is also socially and spatially gendered and a range of situated meanings (Coen et al., 2018), such as narratives of embodied respectability (Green and Singleton, 2006) related to women’s occupation of public space, contribute to a perception of risk for running women (Schmucki, 2012). A survey of gendered experiences of unwanted attention and harassment when running found 46.5% of women, compared with just 9.2% of men, experienced this at least sometimes. In response to the risk of unwanted attention and harassment when running outdoors, female runners explicitly negotiate where they run, what they wear and who they choose as running companions (https://www.runnersworld.com/uk/a775643/running-while-female/).

The growth of running, whether through organised events or personal regimes, can be framed by a neoliberal logic (Ayo, 2012; Lupton, 1997), which shapes an agenda for personal responsibility wherein individuals take it upon themselves to be better citizens (Brown and Baker, 2013, p. 3). This framing, however, can produce over-generalised analyses that miss the nuances, contradictory experiences, and diverse practices through which (un)healthiness is negotiated via fitness orientated cultures (Wiltshire et al., 2018). Participants in parkrun repeated the moral imperative to pursue better personal health through active body projects but simultaneously challenged it through nuanced accounts of embodiment that positioned running as a collectively orientated practice. Wiltshire and colleagues (2017) argue that this collectivity moderates the demands of health responsibilisation and resonates with literature on the therapeutic benefits of being part of something, including being outdoors and being virtuous with others through volunteering (Doughty, 2013; Gatrell, 2013; Muirhead, 2012; O’Brien et al., 2010).

## Volunteering and the emergence of fitness philanthropy

Volunteering is broadly defined as ‘the free giving of an individual’s labour, time, and energy to a larger cause, collective goal, or public good’ (Brown and Prince, 2016, p. 29). Volunteering activities have also diversified greatly from the domain of religious groups and organisations to delivering core public services in a competition to gain contracts to do this with a multitude of other third sector organisations (Milligan and Conradson, 2006). Despite this relocation into public services, the construction of volunteering-in-general as a positive, meaningful set of experiences and activities remains remarkably consistent. This could be due to the conflation of volunteering with ‘giving,’ and altruism in general (Haski-Leventhal, 2009), as well as a substantial literature on the health and psychosocial benefits of volunteering for the volunteer (Casiday et al., 2008; Wilson, 2012, p. 201, although the causal mechanisms for this remain unclear (Jenkinson et al., 2013).

The ‘harnessing’ of volunteers is a familiar feature—and often requirement—of sporting activities, enabling them to remain low cost or free for participants. In England, over two million volunteers supported sporting activities in 2002, mostly within sports clubs (Taylor et al., 2012). This participation and volunteering in organised sports structures has declined since then in favour of more individual and informal activities such as gym membership, running, cycling and walking. These activities are sometimes supported by volunteers, such as in parkrun, where runners sometimes also act as volunteers (Nichols et al., 2016) and described volunteering as a way of ‘giving back’ to ensure the continuation of parkrun as a free and accessible event for themselves and for others (Wiltshire et al., 2017). Similarly, organised walking groups rely on volunteer walk leaders within the community to co-ordinate and lead the walks (Ball et al., 2017; Grant et al., 2017).

The changes in the practices of physical activity towards being publicly exercised and collectively experienced mesh well with volunteerism and afford versatility and malleability that might be harnessed in pursuit of a variety of philanthropic outcomes. Physical activity and volunteering are brought together in ‘fitness philanthropy’ (Palmer, 2018; Palmer and Dwyer, 2019). Fitness philanthropy comprises,

‘consumer-orientated philanthropic solutions to health or social problems that draw on physical activity-based events such as fun runs, bike rides, long swims, epic hikes and multi-sport challenges in which participants seek to raise money for an awareness of a variety of causes, from chronic illness such as motor neurone disease, or cancer, to social issues such as homelessness or poverty’ (Palmer, 2018, p. 198).

Fitness philanthropy offers something rather different to conventional physical activity in introducing an ethical practice into running that is as much other-oriented as self-oriented in terms of affording health benefits. Its newest form, movement volunteering, diverges from the majority of philanthropic running, which tends to focus on raising money for charities through specific events, usually distance-running. Moreover, instead of the site-specific special event, the physical activities are routine, weekly undertakings in the familiar and everyday spaces of the participants. The emergence of this new form of movement volunteering, thus, enables new health-related encounters of bodily movement, ethical commitment, everyday and outdoor public space and the modes of healthy publics that may be emergent.

## Research design and methods

These issues are explored through multi-sited mobile ethnographies in two cities in the North of England with a movement volunteering organisation, the running organisation, GoodGym (https://www.goodgym.org/), which defines itself in the terms of fitness philanthropy,

‘We are a community of runners that combine getting fit with doing good. We stop off on our runs to do physical tasks for community organisations and to support isolated older people with social visits and one-off tasks they can’t do on their own. It’s a great way to get fit, meet new people and do some good.’

GoodGym presents their philosophy, as well as the rationale for their name, in contrast to physical activity through gym membership,

‘We think that gyms are a waste of energy. There are many neglected tasks and people in our communities that need that energy. We want to bring these things together…… We want to rival the success of gyms, getting people all over the world off treadmills and into their communities.’

The activities are threefold: coach runs, mission runs and group runs. Coach runs constitute the innovative heart of the programme in which a runner is matched with a ‘coach’, someone requesting social support and company.

‘Over a million older people in the UK are always or often lonely, some go for months without seeing friends or family. Visiting an older person as part of your weekly run can make a huge difference to their life….We call the older people we visit coaches because they help motivate us to run and they share their wisdom. It’s amazing what you can learn from your coach. Coach runs can fit around your schedule and don’t need to take up more than 20 min of your time per week’.

Mission runs are one-off contributions by individual runners to individual needs, *‘We run to help out older people with one-off practical tasks that they are no longer able to do on their own.’* Tasks can range from small household chores such as changing a lightbulb through to larger needs such as gardening or moving furniture. Group runs are similar one-off contributions, in this case by a group of runners to support local charities with tasks such as decorating, landscaping or sorting out foodbank donations, and often also include a group exercise routine along the way. While the mission and group runs may be one-off, they are organised regularly, weekly when possible, so that participation and moving through the everyday and public spaces of the two cities become a routine engagement for the runners (all quotations from GoodGym’s web-site: https://www.goodgym.org/).

The methodology was ethnographic. Emily, the lead author, volunteered with and participated in GoodGym programmes in two cities in Northern England between June 2018 and September 2019. Emily participated in the mission and group run activities. The coach runs involve a relationship of trust built up over time, usually with older people, which we considered not ethically open to ethnographical research beyond the reported accounts of the runners. The specific methods combined mobile participant observation and semi-structured interviews, including the ‘go-along’ method (Carpiano, 2009; Evans and Jones, 2010; Garcia et al., 2012; Kusenbach, 2003). Participant runners also selected photos they had taken as part of the GoodGym activities, wrote a reflective commentary on their experiences and discussed this with other participants during a knowledge exchange event held at the end of the fieldwork.

The participant observation with the two GoodGym groups included 41 volunteers, 2 employed co-ordinators, and 3 members of charitable organisations for which GoodGym carried out tasks. During the fieldwork, Emily took part in 80 different GoodGym activities with a volunteering element, each lasting 1–3 h, as well as a dozen additional social activities and races. GoodGym also has an online platform for logging runs and activities, sharing run reports and photos from the activities, which enabled keeping up with the participants’ activities remotely. The fieldwork data comprise extensive fieldnotes from participant observation, mobile ‘go-along’ interview recordings with other volunteers in the programmes, and photographic visual data. On-going analyses of data during the fieldwork generated further reflective researcher notes on the primary ethnographic and interview data, raised further discussion points with research participants and informed a knowledge exchange event held at the end of the fieldwork and in which themes of public space, moving together, inclusivity, and sociability were explored.

The project was subject to the procedures for ethical scrutiny and approval of Durham University, which are fully compliant with the guidance of UKRI. All participants received full information on the purposes of the research and the use of the research data, and agreed to participation in the study through a signed consent form. In the presentation of results, all names have been changed.

## Engaging and participating in public spaces

Taking part in the movement volunteering opportunities enabled volunteers to participate actively in public spaces. The groups worked on, used, and moved through these spaces in different ways, bringing about a variety of place-based interactions with people, materialities and affectivities or atmospheres. Movement volunteering often brought a sense of being part of the general rhythms and interactions of the cities’ public life and public space. In and through these relations, the moving-volunteering bodies generate therapeutic gains not only for themselves but for a range of intended beneficiaries and unrelated passers-by and for the material and affective environments of the public spaces. These generative encounters of people, place and things, which are both formed and necessitated in movement volunteering and in which wellbeing is both an emergent, relational and situated effect and a collective one (Atkinson, 2013), may be seen as constituting ‘healthy publics’ as conceived by Hinchliffe et al. (2018).

We present our findings in relation to three of the different constituencies who, intentionally or otherwise, participate in the formations of such healthy publics: volunteers; passers-by; space and atmosphere. We then identify processes of formation of healthy publics in terms of visibility and spectacle, sociality, and merging mobilities.

## Volunteers

### The ‘doing good’ element

Those joining as volunteers were a key focus in researching motivations in joining GoodGym, experiences as runner-volunteers and accounts of the therapeutic benefits accrued through the activities. Volunteers revealed a range of motivations for joining GoodGym and while there were many comments, explicit and implied, on the element of doing good, overall this was not stressed as much as might be expected given the centrality in GoodGym’s ideology.

GoodGym’s optional membership donation are cheaper than joining a conventional gym but nonetheless more expensive than joining a conventional running club. People do not, it seems, join GoodGym merely as a cheap exercise option. Time commitments are flexible, but structured and regular. Neil, for example, flags ‘the doing good thing’ as a strong motivation, but equally stresses the attraction and manageability of regular scheduling of activities given he was looking for something to do:

Neil: I have a few free evenings and on the weekend which I usually spend just standing around at home, or sitting at home…so it’s no big sacrifice, there’s not something better that I could be doing instead of it. Because it’s a regular weekly thing I find it quite easy to commit to. And because it involves other people and especially the doing good thing I find it provides a strong motivation

**Fig. 1:**
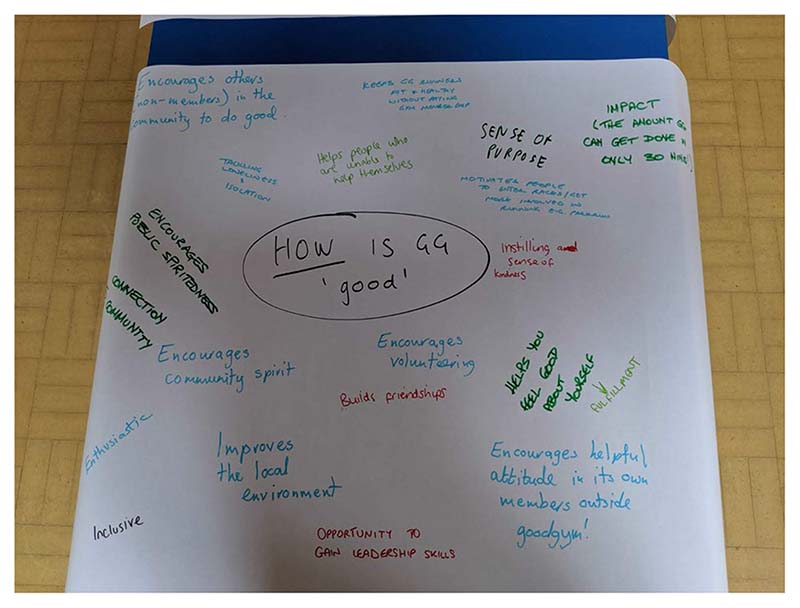
‘What makes GoodGym good?’. Results of brainstorming by participants at the Knowledge Exchange Event in August, 2019.

Other incidental comments reveal motivations of sociability and fun, the commitment to older people, and, the relative ease of joining compared with the application process for many other volunteering opportunities, which one participant described as similar to applying for a job. Participants at the Knowledge Exchange Event in August, 2019, brainstormed responses to the question ‘what makes GoodGym good?’ and key points are recorded in Fig. 1. The ‘good’ qualities of GoodGym fall into three categories. Individual benefits to volunteers include keeping healthy through running, building friendships, having a sense of purpose and feeling good about yourself. Mediating benefits include encouraging an attitude of helpfulness beyond GoodGym, encouraging volunteering and generating enthusiasm and inclusiveness. Finally, benefits to the wider community include specific goals of helping where people cannot do things themselves, addressing loneliness and isolation, improving the local environment, and some broad cultural goals such as building public or community spirit, building connectivity and instilling a sense of kindness. Talk of ‘doing good’, however, may be culturally uncomfortable in the United Kingdom, which may underwrite the relative absence of volunteers expressing themselves directly in this mode. When Emily encountered someone happy to talk in this way, she logged her own discomfort in her field-notes,

I ask how the community mission was set up and he says Taskforce people can set them up and suggest them. I say it’s amazing how many different things people are involved in - means there is no shortage of tasks! Then he said something along the lines of ‘well, good people do more good things’ which I thought was an interesting comment; do good people do good things? Does doing good things make people do more good things? Does doing good things make you a good person? [Field-notes, 29.11.2018]

### A different way to run

The involvement in public space by the GoodGym runners was not only through the volunteering actions to help the environment, local charities and other not-for-profit organisations. It also came about through moving together as a group, moving outside and enjoying the physical benefits of running. More particularly, the value of GoodGym for runners is less about the embodied gains per se and more about the ways in which that embodiment takes place; finding a different way to run was a common reason for people’s participation in GoodGym. Runners who had previously run marathons said that they wanted a different sort of goal. Runners with injuries wanted a way back into running, while those new to running saw the additional purpose in GoodGym runs as an interesting way to take up the activity. GoodGym’s practice of running in a mixed ability group, thus, facilitated different experiences through a culture of running characterised by togetherness rather than competition. Gavin, for example, describes himself as a regular runner; he used to be in a running club, he does parkrun, has run marathons and other races, and regularly uses Strava to record and track his performance. His aim now is to ‘enjoy running more’ through a less demanding form of training:

Gavin: One of the reasons I joined GoodGym was just to enjoy running a bit more, so obviously you’re training for marathons and stuff, it takes a lot of your time, whereas this is a lot more relaxed and you still get a good few miles in.

Interviewer: So you still see it as a run…

Gavin: Yeah even though it’s not obviously training very hard everyday I still see it as part of my training, it’s still extra miles for my legs…

Interviewer: Mileage?

Gavin: Yeah its mileage! If it goes on Strava, its all worth it! (Laughs)

While Gavin indicates that, for him, getting the miles in was important, the accumulated mileage of the distance run is unifying rather than competitive. The focus on building up the distance run is done by each runner setting their own targets and their own speed.

### Running as a mixed level group

Other runners in GoodGym commented on becoming more sensitive to the different levels of fitness when running as a group. The action of moving together with others of different fitness levels enabled an increased understanding of variation in embodied and everyday experiences. Bernadette, for example, related an occasion when she was chatting with someone on a group run. For her, the pace was comfortable and she chatted away easily but she noticed the heavy breathing of the other runner. She said it allowed her to appreciate more the efforts of others in the group even while becoming aware of her own fitness level. Zak, who began GoodGym as a less experienced runner, experienced the same characteristics of mutual appreciation, support and cameraderie that offered a comfortable culture for starting out as a runner, but extended to a much wider range of inter-personal engagements:

‘GoodGym has directly and indirectly lent me the perspectives of other runners and volunteers from different backgrounds, on an equal footing, from which I like to reflect on my own attitudes and perspectives. I am constantly brimming with fun/useful ideas as the result of conversations had within the group. However, I don’t feel inclined to compare myself to others when I am in a setting with GoodGym runners, which speaks to the level of comfort I feel in the cooperative environment created, the growing bonds of trust and friendship, and the caring sincerity of the runners I share my time with. As a result, I feel able to be myself openly, and many of my preferred personal traits can find expression with greater ease than in other contexts. I wish to continue to build my contribution to the group because I am inspired by the effect GoodGym has on me. I was intrigued to gain a sense of this effect in my very first run, and it has been positively reinforced at each GoodGym-related activity since.’

[Zak, Knowledge Exchange Event, 4.8.19]

Zak’s self-analytical narrative here highlights the comfort and inspiration that he feels as part of the group. Being able to be himself and share ideas was a valued element for him. He attributes this to being on an equal footing with the other runners, and not feeling like he has to compare himself with them.

**Fig. 2:**
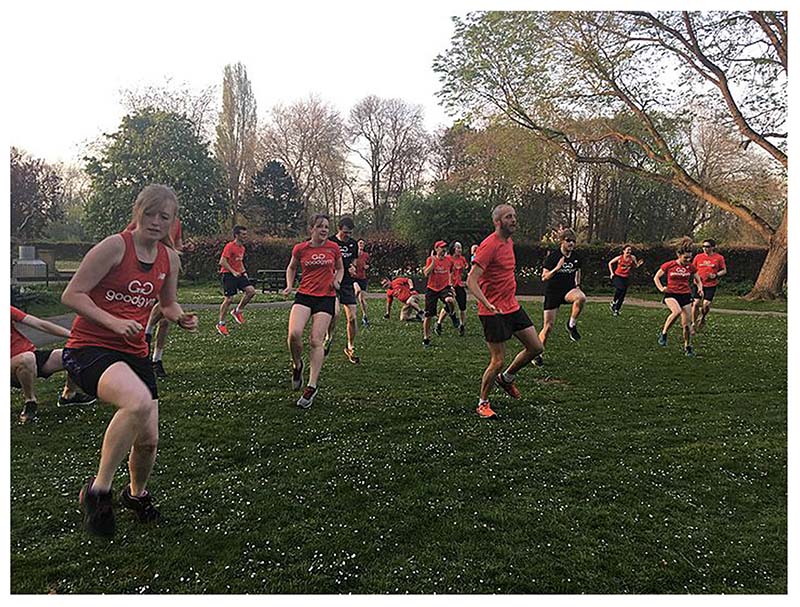
Togetherness photographed by ‘Phil’. An outdoor fitness session involving synchronicity of movement through dance with each other and with the music.

The embodied benefits of running within a culture that was comfortable and less competitive, are complemented by the perceived benefits reported of being and moving together. The examples above partially reflect this, although attention is drawn to acknowledging and respecting difference. Moving as a group while running and in the GoodGym activities draws attention to the good feelings generated by togetherness itself, through a synchronicity of movement. Phil, for example, provided a photograph to the Knowledge Exchange of an outdoor fitness session involving synchronicity of movement, to each other and to music when dancing (Fig. 2). Phil wrote in the caption to the photograph:

‘Togetherness: One of the many fitness sessions to music - nearest I get to dancing and symbolises the togetherness of every GoodGym Group run for me’

[Phil, Knowledge Exchange Event 4.8.19]

### Visibility

The togetherness that is experienced as supportive, social and synchronised takes place outside in public spaces, and the group running of GoodGym makes the volunteers particularly visible within their movement settings. The GoodGym runners, in their matching red t-shirts and running as a pack in urban spaces, are particularly visible, and Emily captures an example of this in her notes and transcript from a go-along interview with Gavin. ‘It is the Monday night group run and we are running in a group across a bridge. It is in the run up to Christmas and some people are dressed in Christmas outfits. The cars are zooming past and it is freezing cold. I am conducting a go-along interview with Gavin and we are discussing what it is like running in a group.’ He says:

Gavin: ‘Yeah cos obviously we’ve got the same interests…but different paces and distances…there’s that social aspect, that knowledge that everyone around you is doing the same thing…I think it’s much easier training in groups…you will have to train by yourself obviously, you have to have that ability to, you know, stay in a group…if you’re running races you know, you’re not going to be doing that by yourself…you’re gonna have people that are faster than you, slower than you…yeah so running in groups is good for that…

(Cars beep at us as we cross the bridge …)

Gavin: I mean like that…that makes me laugh right…people driving past thinking what the hell are they doing and we are just running along like…

(More car beeps)

We both laugh.’

The dynamics of the place through which they were running allowed Gavin to show, as well as tell, what he enjoyed about running together in a group. He begins by highlighting the performance advantages of running in a group. The cars beeping, however, as they ran along the bridge, showed additional elements of running in a group that Gavin enjoyed—the responses from people passing by, and the general spectacle of it. These reactions from those not involved in the group runs were commonplace, and the runner-volunteers viewed this as a meaningful element of the group run. This was confirmed at the knowledge exchange workshop in a brainstorming exercise on the different relationships involved in the movement volunteering programmes in addition to those explicitly set up with potential beneficiaries which generated,

‘with random passers-by who say ‘thank you” and ‘with people who shout at you to ‘go faster” and also ‘with all the people who ask you what GoodGym is and what you are doing’ [Group Brainstorm, Knowledge Exchange Event 4.8.19].

These fleeting relationships were clearly viewed as an integral part of running and a valued element of the activity as a whole. The efforts made by GoodGym as a fitness philanthropy organisation to contribute locally also garner positive feedback that is affirming and can at times be not only unexpected but also reassuring to the runners’ own anxieties about what they are doing:

‘On our way back to the start location, we navigate around a couple of rough sleepers who are walking slowly along with big bags and sleeping bags. I always feel a sense of awkwardness and helplessness when we run past homeless people on the street wearing our GoodGym t-shirts - to me, people on the streets probably need help most urgently, and I wonder about the kind of good we do and the way in which our good deeds actually impact people who need it most immediately. They move to the side as we run past and I hear one of them saying to his friends in slurring words, let them past, let them past they run and they do good stuff, before shouting after us words of encouragement. We turn around and wave back to acknowledge that we heard’

[Fieldnotes, 24.09.18].

## Passers-by

The benefits felt by the volunteers in doing-good, in doing-good together through synchronicity, achievements, or supportive respect, and the enjoyment of the public encounters with passers-by are all complemented by the benefits that accrue to others. These others include the intentional beneficiaries such as the ‘coaches’ that the GoodGym runners visit. These also include, however, unintentional encounters with passers-by in public spaces, such as with those beeping their car horns described by Gavin, who directly interact with the volunteers in various ways.

### Encounters

**Fig. 3:**
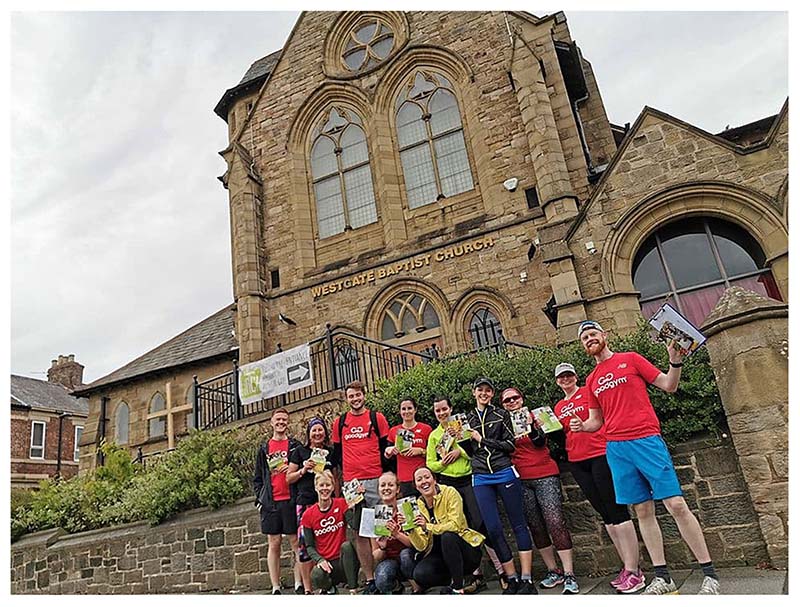
Encounters on the street. A GoodGym running group photographed for them by a passer-by.

Passers-by play an integral role in forming memories and experiences of particular tasks. In his photographic submission for the knowledge exchange event, Callum shared a photograph of a GoodGym running group that had been taken by a passer-by. The passer-by took his role as photographer very seriously and spent time getting the perfect shot (Fig. 3), which Callum appreciated and remembered:

‘This photo is very artistic. Shot at a Dutch angle, a passer-by lay on the floor until he had perfected the shot’ (Callum, KE event 4.8.19)

For Callum, the interactions with a passer-by made the activity all the more memorable and meaningful for him, and what-is-more, from the degree of commitment made by the passer-by as photographer, they also enjoyed the encounter.

Passers-by sometimes responded to activities in comedic or mocking ways. Children in particular, who are already playing outside, often with bikes and scooters, saw an opportunity to make fun,

‘Meg is just finished explaining the session when we arrive. We are in a brand new play park with climbing frame and lots of different structures. Meg has designed a kind of circuit round them all but we are not to worry about doing it wrong—the aim is really just to ‘play’ on the equipment. I start with step ups on to steps of different heights and then move on to the climbing frame, which I try to navigate round without my feet touching the floor (or knocking anyone else off). I notice that a couple of kids have jogged along the last bit with us and are mimicking us’. (Fieldnotes, 6.8.18)

This engagement, mocking notwithstanding, effectively blends the passers-by into participants in the GoodGym activities, as when residents in supported accommodation for vulnerable young people encountered the volunteers holding a fitness session at the site,

‘A couple of the residents join in the fitness session, half mocking us—grinning, laughing. One of them breaks down into a coughing fit, and a couple of the GoodGymers chuckle. They seem a bit out of it. One of them keeps shouting knees up, that’s it, higher - mimicking the role of the fitness instructor, to the amusement of his friends’ (Fieldnotes, 24.9.18).

People also voiced commentaries without any direct physical involvement on what we were doing, such as those outside the pub shouting at the group, *‘smiles not miles!’* (Fieldnotes, 24.09.18), or those yelling at some of the female group runners, *‘keep running, it’ll make your bums nice!’* (Fieldnotes, 18.06.18). In these encounters, the spectacle of the group movement drew other people’s attention and prompted their own actions of mocking, mirroring and exaggerating the movements of the volunteers. Emily noted that at times this felt like a form of impromptu audience participation, which highlighted the performative element of their activities but also altered the status of passers-by from passive ‘extras’, or obstacles to navigate round, into active participants.

### Opportunities

Being out-and-about as part of movement volunteering presented opportunities not only to engage with others but also on occasions to provide ad hoc assistance beyond the remit of the task at hand. Examples included helping jump start a car or supporting someone who had fallen off their bike. Running to a mission for an older person, one of the GoodGym runners even managed to catch an escaped greyhound and stopped it from running into the road. As all GoodGym activities are written up in the form of a ‘run report’, these one-off, instinctive and opportunistic actions while on the move could be acknowledged, shared, and celebrated.

## Space and atmosphere

The activities of movement volunteering, the experiences of the volunteers and their extension to others sharing the spaces in which the movements are playing out all interact to co-constitute those spaces and places in both tangible, material ways and in fleeting, atmospheric ways.

### Tangible and material effects

**Fig. 4:**
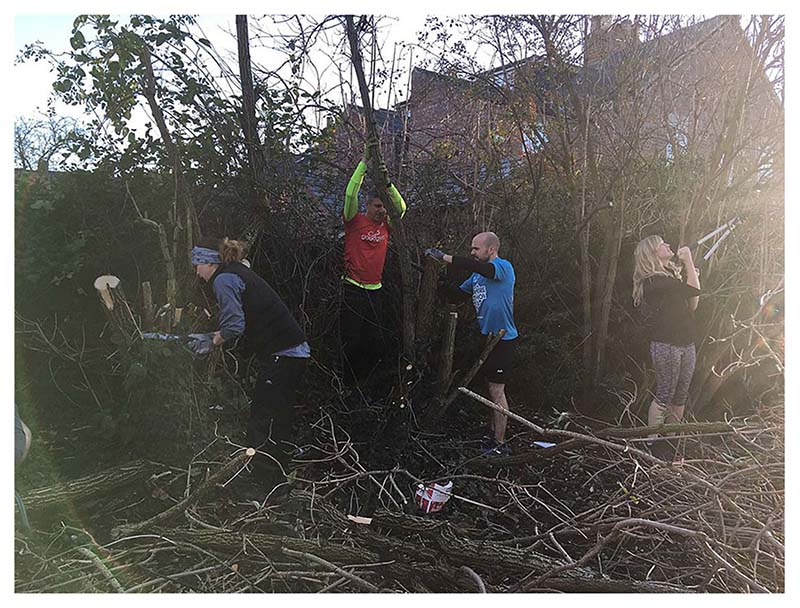
Making a tangible difference photographed by ‘Brad’. A GoodGym community project to clear and manage the environment.

The GoodGym group activities often produced material or tangible effects and these, in turn, resulted in good feelings for the volunteers from their evident achievements. The volunteers often captured their achievements visually through photos to show the difference made to particular places through environmental or community-based actions. Brad, for example, provided a photograph to the Knowledge Exchange Event (Fig. 4) and commented on the benefits that attend making a substantial and visible difference through a GoodGym community project, in this case an environmental scheme:

‘Making a visible difference: Whilst I loved doing previous tasks, this was one of the first tasks where I could see we made a massive visible difference and felt really good about it…’

[Brad, Knowledge Exchange Event 4.8.19].

Such group interventions afforded one way in which people began to feel connected not only to the places where they had volunteered, but also to the wider philosophy of the GoodGym movement as the tasks undertaken became memorable.

### Fleeting atmospheric effects

The movement of the GoodGym runners through urban streets and spaces prompts reactions and interactions variously characterised by curiosity, derision and sexism, positivity and fun. The effects of the programmes thus impact the ambient atmosphere of the spaces and places through which the volunteers are moving. The operations of fitness philanthropy in public spaces, and the specific adoption of community projects by GoodGym make their activities particularly noticeable to residents and passers-by.

Alongside the joking and incredulity that could accompany the GoodGym group run, one occasion of community activity served to arouse suspicion from onlookers. It was a cold night in March and the task was to rip up wetpour at a local play park. This excerpt is from Emily’s fieldnotes:

‘Once sections had been ripped off, they were piled into wheelbarrows and wheeled across to the edge of the park, where a mountain of discarded wetpour was beginning to form…… On a trip to the wetpour mountain, I notice a police van has pulled up outside the play park and a policeman is talking to one of the group. I listen to the person trying to describe what we are doing here—there’s a man from the council here who is in charge, they say. The policeman seems satisfied. Apparently, some concerned neighbours had called in, quoting suspicious activity in the park. We fall about laughing when the policeman leaves for another job—what kind of gang wears matching t-shirts and headtorches? Someone says, now we are literally ‘BadGym”

[Fieldnotes, 11.3.19].

This is, effectively, a conflicting encounter of public spiritedness whereby GoodGym’s voluntary activity aroused suspicion from a well-meaning neighbour, who, under the darkness of the evening, spotted a hub of unexpected activity involving handsaws, spades, and other tools. The darkness of the evenings changed the feel of the group runs, prompting the use of headtorches and flashlights to see. This generated jokes about what work done might look like in the light of day and the feelings of acting like an undercover operation.

In contrast to the potential for suspicion, the runner-volunteers noted that their presence on the streets, and most often in the evening, might actually improve the safety of an area. At the knowledge exchange, the participants wrote down all the groups, places, and spaces that are involved in or that benefit from the movement volunteering charities. Someone simply wrote down ‘*the street*’, which sparked conversation in the group. The contributor explained that the GoodGym runners literally help ‘the street’ by maintaining paths and other aspects of the material environment. They also, however, inhabit the space of the streets with their bodily presence. Where residents or those moving along a street feel neither safe nor connected to an area, the sight and presence of a group of people involved in making it better not only makes the street safer through the presence of more people, but also makes it feel safer through the care being given to the space.

## Constituting healthy publics

There is an assumption in conventional exhortations to physical activity that exercise is disconnected from other people in that we do things with our bodies that are not particularly useful or engaging for anyone except the exercising individual. Running on a treadmill serves as the metaphorical exemplar of physical exercise that goes nowhere, and the inspiration for the GoodGym concept for CEO Ivo Gormley, as he explains at a TEDx event (https://www.youtube.com/watch?v=jS7tPx2vZRU). Bodily movement in everyday spaces connects people to places and with therapeutic outcomes (Gattrell, 2013; Doughty, 2013). Forms of embodied travel, walking, running, cycling and so forth, constitute therapeutic mobilities in and of themselves, but the spaces through which bodies move also configure the therapeutic experience (Gattrell, 2013). This is strongly evidenced for being outdoors in green and blue spaces (Bell et al., 2018) to the extent of being viewed as intrinsically life-affirming. This may emerge from an’intense embodiment’, in which ‘a positively heightened sense of corporeal aliveness’ occurs (Allen-Collinson and Leledaki, 2015, p. 467) and in which spirituality and spiritual healing may feature as core components (see Gesler, 1993; Priest, 2007). Our moving bodies can be sites of creativity (Tainio, 2018), pleasure (Throsby, 2013) and resistance (Bajič, 2014). Bodies are creative through ‘playful and creative experimentation can bring new ingredients into an established practice’ (Tainio, 2018, p. 12). A heightened kinaesthesia in exercise may open up a positive, sensorially enhanced space affording experiences of unexpected pleasure, as described by Throsby in long-distance open-water swimming (Throsby, 2013, p. 13). The moving body can also offer a site of resistance to normative or conventional practices, such as counter-movements to the high-tech filled running culture through barefoot running and everyday walking (Bajič, 2014).

The benefits of doing physical activity with others, as well as outside, has also been well documented, although Hitchings and Latham (2017) argue that those with a primary focus on their running value more a ‘light’ sociality, ‘a range of socialities that variously helps runners remain on task, provides distractions, and offers a sense of being involved in the communal rhythms of the neighbourhood’ (Hitchings and Latham, 2017, p. 6). Others have observed how moving and talking together within a group, outside, promotes wellbeing, such as in the ‘shoulder to shoulder’ support described by participants in a women’s breast cancer recovery walking group (Ireland et al., 2019) or the conversational and non-competetitive camaraderie seen in a group for older adults (Copelton, 2010). The dynamics of moving together enable ease of social interaction, and participants in Ireland et al’s study described ‘flows’ and ‘shifts’ of conversations that were enabled by the physical formations of the group as they walked. Running together similarly facilitates brief interactions establishing commonalities and bonds through the ‘small stories’ of asking for advice, validation and information (Griffin and Phoenix, 2016). The moving body is thus a medium of connection, and when it moves outside, it becomes a way for people to connect with places and others in shared spaces.

These relational and situated accounts challenge simplistic models of the benefits of exercise as purely physiological and make visible the embedding cultural imaginaries of meaning, but they do still focus on the benefits of physical activity for the exercising individual body. Fitness philanthropy, especially in the form of the movement volunteering explored here, does something rather different in its practice by upholding the virtues of health typically claimed within exercise rhetoric while inhabiting spaces for connected exercise in which our bodies become dynamic sites and agents of moral and virtuous action. Our study of GoodGym expands this understanding and describes some of the many ways that the movement of bodies in public spaces, and the movement of bodies intending to be useful, generate far greater therapeutic potentials than for the individual moving-volunteering body alone. The resonance with and contribution of these new practices with the notion of healthy publics (Hinchliffe et al., 2018) inheres in the shared efforts towards provoking and facilitating a shift in how we think about health-related interventions. This shift expands a focus on individuals or passive recipient publics to embrace a more dynamically and relationally constituted set of conditions for health and engagement with a range of constituencies, from human through other life forms to inanimate materialities and affects. The practices of fitness philanthropy described here not only challenge dominant thinking in terms of individual benefits, but, in themselves, work to constitute a mixture of transient and more enduring formations of healthy publics comprising people, things, spaces and affects. The formation of these dynamic, multiple and shifting healthy publics emerge through the complex intersections of several processes. We draw particular attention in this section to the centrality in the new fitness philanthropy practices of visibility and spectacle, sociality and merging mobilities.

The most striking feature in relation to the formation of healthy publics through fitness philanthropy programmes is, arguably, that they play out in a highly visible public space. While interactions with passers-by have been observed in a smaller way in walking groups (Morris et al., 2019), the GoodGym runners, wearing the team t-shirts and often group running in the evening, are a highly visible presence on the city streets and it is this visibility and sense of spectacle that draws unintended beneficiaries into engagement and participation in an emerging healthy public. The repeated examples of passers-by acknowledging the sight, presence and activities of the fitness philanthropy volunteers on the streets attest to the spill-over effects of the programmes beyond merely the volunteers and the formal participants in ways, which generate far wider benefits to people, places and atmospheres. The accounts of the volunteers make evident the importance of visibility in building explicit connections with others sharing the same space. This visibility, moreover, is often one of spectacle in which the actions of the volunteers, and sometimes the participants, combined with the materialities of clothes and tools, effectively generate a show, a form of entertainment and enjoyment for others.

The importance of visibility and spectacle overlaps considerably with the importance of sociality. The fitness philanthropy activities have at their core an understanding of the value of being and acting together, of providing social connection and interaction for those who may otherwise feel rather isolated and of contributing time and energy to local projects that benefit territorially defined communities. Social interaction in itself, however, does not automatically equate with beneficial or therapeutic wellbeing effects. A particularly notable characteristic of the social interactions brought into being through the movement of the GoodGym runners is their infusion with good-will, humour and general ‘bonhomie’. GoodGym volunteers express a sense of shared concern and care not only for the people and projects they help through their activities, but also for one another. Others have argued that the emergence of a sense of selfcare through movement can be construed as a form of resistance to disciplinary approaches to physical activity (Lloyd et al., 2016; Markula, 2004) and we suggest that the ‘shared care’ seen in GoodGym can be interpreted in the same way.

The ways in which fitness philanthropy brings bodies together to move generates benefits from what we can understand as merging mobilities. For the GoodGym volunteers, the group runs bring together bodies with different levels of fitness and abilities for running. The accounts of the volunteers specify how they appreciate their own growth in awareness and attunement to other bodies through a running culture that is less competitive and more cooperative. GoodGym also holds collective activities that do the opposite by enabling awareness of the similarities of bodily capacities as when volunteers are working together on a community-based project or moving in synchrony with each other and with music on a fitness activity. The movement of the volunteers in and through the sites and spaces of the cities facilitates interactions with those spaces such that they effectively mutually constitute one another. Direct effects from the programmes include material changes through environmental and community projects, improved street safety through their presence in those spaces and a cheerful street atmosphere through the spectacle and enjoyment generated by the visibility of the runners.

We have in this paper endeavoured to explore how moving and doing good at the same time, through entangling of practices previously seen as distinct, effectively reworks the implicit model of health promotion as by the individual for the individual to one embedded within complex assemblages of people, things and places. We have detailed some of the ways in which these reworkings of health promotion and publics play out and emphasised the centrality of visibility and spectacle, of sociality and of merging mobilities as forming healthy publics along the lines envisaged by Hinchliffe et al. (2018). Our study foregrounds important and unintended spill-over effects from the new practices of movement volunteering in everyday spaces that not only reworks but extends our conventional understandings of exercise, health promotion and the formation of healthy publics. The healthy publics brought into being through an everyday fitness philanthropy share with existing conceptualisations a central health concern, in this case physical activity (Healey, 2019), an intentional purpose, albeit one with a duality of health and social purposes, and emerge through a multiplicity of situated and collective relations and practices. Our participants’ descriptions of their GoodGym activities through mixtures of embodied, moral, affective, technological and social experiences resonate with the existing emphases on dynamism and multiplicity in the formation of discursive, affective and networked publics (Lünenborg and Raetzsch, 2017; Lünenborg, 2020; Rock, 2017; Stephansen, 2016). The elements that coalesce into a healthy public through movement volunteering, however, comprise more than the self-defining constituencies of interest of the GoodGym volunteers or their target beneficiaries, comprise more than the specific sites identified for mission or group run actions, and comprise more than the words of voices, discourses or debates of formal and informal health debates (Hinchliffe et al., 2018; Mahony and Stephansen, 2017). The practices of fitness philanthropy emphasise the everyday spaces and times of the city and affirm existing work that reimagines a public from a given, relatively static and passive recipient of interventions into a constellation of multiple, dynamic and emergent actors in health. This spatial emphasis affords additional conceptual value by extending the concept to embrace and value the generation of wider place-based encounters that, although fluid and transient, nonetheless leave beneficial effects in their wake.

The next steps towards a more theorised account of healthy publics includes exploring the ways that these diverse participants within a dynamic healthy public, whether intentional actors, human, situational or affective, may be effectively engaged more directly into the formulation of approaches in health promotion that are both sensitive to these dynamics and better able to build on their strengths in enabling more flourishing societies.

## Data Availability

The data sets generated and analysed for the current study are not publicly available as: (a) the data are still in process of analysis for a doctoral thesis and under embargo; (b) the data were generated under guarantee of confidentiality and many are sufficiently individual to disclose identity. The holder of the data set, ET, first author, will willingly discuss the data, the analysis and interpretation with researchers working in a similar area.
